# Surveillance of C-Allocation in Microalgal Cells

**DOI:** 10.3390/metabo4020453

**Published:** 2014-06-19

**Authors:** Heiko Wagner, Anne Jungandreas, Andrea Fanesi, Christian Wilhelm

**Affiliations:** 1 Universität Leipzig, Institute of Biology–Plant Physiology, Johannisallee 23, 04103 Leipzig, Germany; E-Mails: hwagner@uni-leipzig.de (H.W.); andrea.fanesi@uni-leipzig.de (A.F.); cwilhelm@rz.uni-leipzig.de (C.W.); 2 Department of Computational Landscape Ecology, Helmholtz Centre for Environmental Research–UFZ, Permoserstraße 15, 04318 Leipzig, Germany; E-Mail: jungandr@rz.uni-leipzig.de

**Keywords:** FTIR spectroscopy, algae, biomass, composition, metabolomics, chemometrics, *Chlamydomonas*

## Abstract

When microalgae are exposed to changing environmental conditions, e.g., light-dark cycles or oscillations in nutrient availability (CO_2_, nitrogen, phosphate or silicate) they respond with metabolic changes in the carbon allocation pattern. Short time regulations in the time range of few seconds to minutes can be mirrored best by mass spectroscopy based metabolomics. However, these snap shots do not reflect the alterations in the carbon flow to the cellular macromolecules like protein, carbohydrate or lipid. In this review it is shown how the combination of FTIR spectroscopy and Chla-*in-vivo*-fluorescence based electron transport rates can reveal changes in the metabolic flux rates of carbon during a shift of the environmental conditions. The review will demonstrate in which time range FTIR spectroscopy can deliver significant information and how FTIR spectroscopy data can synergistically support metabolome analysis by mass-spectroscopy.

## 1. Introduction

Importance of C-allocation in biotechnology and ecology

Recently, microalgae have attracted increased attention in the field of biomass production and biosynthesis of feed stock components or high valuable products like polyunsaturated fatty acids, antioxidants, pigments like carotenoids which can be used for food colorants, *etc*. The most challenging approach to increase the productivity is not only to develop photo-bioreactors which offer optimal conditions for algae growth, but also to increase the yield of a special product [1]. Since an increasing number of algal genomes are now available and genetic transformation protocols have been published to apply system biology for the green alga *Chlamydomonas reinhardtii* [2], but also for *Ostreococcus tauri* [3], the diatom *Phaeodactylum tricornutum* [4] and for *Nannochloropsis sp.* [5], there is increasing need to develop quantitative methods to measure the carbon flow from photosynthetic primary products into the component of interest. Typically, metabolic engineering is used to channel the carbon from sugar to lipid, starch, protein or secondary products like pigments. For this purpose the cells are genetically transformed either by inhibition of competitive pathways or by stimulation of the pathways that lead to the product. After having checked integration, expression and translation of the new gene, the simplest test to look for the metabolic effect of the transformation is to measure the content of the compound of interest during growth. However, genetic transformations of microalgae can also alter the overall activity of the cells leading for example to lower photosynthetic performance. In that case it is difficult to decide if the concentration of the product is influenced due to inefficient metabolic engineering or due to the impaired photosynthetic activity.

To understand changes in metabolic fluxes in the cellular network of carbon allocation the method of choice is metabolic profiling using carbon isotopes in pulse chase experiments. However, this methodology is time consuming, costly and can be improved by well-defined sampling, when the time points of the metabolic switch from one pathway to another can be identified (see below). Since a metabolic profile is a snap shot of the metabolic state at a given moment, the changes in the carbon allocation network can be detected only by comparing snap shots from different time points. However, cells grown in a light dark cycle or in batch culture change the metabolic profile also in response to the environmental conditions, internal clock oscillations or reorganize the metabolic network during a transition period to turn back to the initial state. This problem can be solved if the time scale of metabolic dynamics can be measured by fast and easy to handle methods which can be used to find a well-defined sampling. Here we present a new method of choice involving the application of Fourier Transform Infrared (FTIR) spectroscopy, which delivers information about the relative size of the cellular macromolecular pools that might change in response to metabolic engineering (e.g., protein, carbohydrate and lipid). A second problem of metabolic engineering is to measure cell internal carbon flux rates. A metabolic profile delivers at best relative concentrations of a set of metabolites but it does not tell the kinetics of changes in the pool size of the components identified. This is especially important for those metabolites which occur in different compartments in different activated states, e.g., phosphorylated *versus* non-phosphorylated or oxidized *versus* reduced form. Here again FTIR spectroscopy of intact cells can be used to measure flux rates between and into the macromolecular pools, but with much less molecular resolution than metabolic profiling. The paper gives an example for this approach.

## 2. Methodology of FTIR Spectroscopy and Statistics in Spectra Interpretation

**Figure 1:**
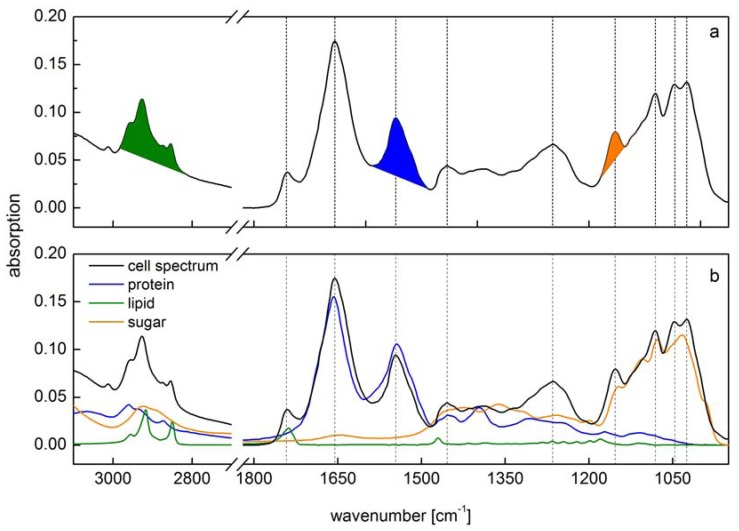
Comparison of two different methods for spectra interpretation. (**a**) Peak integral quantification of macromolecule contents [10]. Peak integrals have been marked (green: lipid; blue: protein; orange: carbohydrate); (**b**) Spectra reconstruction by reference spectra (green: lipid; blue: protein; orange: carbohydrate) of the same cell spectra according to [13]. Vertical lines indicate main peaks of the cell spectra.

Traditional analysis of C-allocation or protein:lipid:carbohydrate ratios by means of biochemical methods require relative huge amount of biomass (in the mg range) and due to their time consuming procedure they are not suitable for high throughput measurements. However, analyzing the macromolecular composition that consider the whole pool of cellular compounds need new approaches to become faster and decrease the amount of sample necessary to measure. UV-VIS spectroscopy itself has been established and accepted for quantifying a wide range of cellular compounds (e.g., pigments in algae cells). Far less attention has been given to FTIR spectroscopic methods, although a single spectra can be obtained in the range from less than 500.000 cells down to single cells compared to several mg necessary for standard biochemical methods. FTIR spectroscopy was used for the first time on phototrophic organisms by Kansiz *et al.* [6] to discriminate different cyanobacterial strains but not to quantify the biomass composition. However, this work initiated a variety of different methods and applications using FTIR spectroscopy to analyze cell biochemical features in phototrophic prokaryotes and algae. Within this review we will focus on the determination of biochemical cell composition and quantification of cell constituents (e.g., lipid content). Some key works in the last decade showed the applicability of IR spectroscopic methods for quantifying cellular protein, lipid and carbohydrate contents in phototropic prokaryotes and algae. The methods in general are based on the absorption of IR light by specific chemical bonds reviewed in Movasaghi *et al.* [7]. By absorbing IR radiation changes in dipole moment of chemical bonds are required. This leads to the generation of specific vibrational features e.g., bending and stretching at different wavenumbers due to the different frequencies (energy value) of IR radiation [8]. Therefore, a single macromolecule has a very characteristic absorption spectrum in the far red region of the light spectra composed of different absorption bands like amide I, amide II and amide III band for protein. These absorbance features of single macromolecules result in a complex cell spectrum (Figure 1) which is composed of all IR active biochemical cell components. Since vibrational bands of macromolecules are strongly overlapping each other, the interpretation of cell spectra is one of the crucial challenges in IR spectroscopy.

Several methods have been developed so far, ranging from the use of peak ratios [9] over the determination of peak integrals [10,11,12] up to a complex mathematical fitting procedure of spectra using single reference spectra for a multiple linear regression analysis [13]. Different extinction coefficients for each chemical bond makes the interpretation even more complicate [8]. Therefore, it has to be distinguished between semi-quantitative changes of biochemical components on the one hand and absolute quantitative determination on the other hand. For semi-quantitative determination of changes in C-allocation like altered ratios of protein per lipid it may be sufficient to use absorbance peak ratios [14,15]. Furthermore, ratios of band integrals can be used to monitor for example the relative lipid content [12]. These methods can be adequate for most of the applications e.g., monitoring cultures for responses to nutrient limitations (see below), but cannot provide absolute values. A semiquantitative approach for the determination of the macromolecular pools in species of different algae strains has been introduced by [16]. Within this study the cellular protein contents were measured by biochemical methods to correlate the ratio between the FTIR absorbance of the pool of interest and that of proteins [16]. Other applications that need more precise information on absolute quantitative amounts of protein, lipid and carbohydrate require more complex examinations. Here, two different main methodical approaches are established. The use of band integrals calibrated by external single standard substances for quantitative analysis of biomass composition has been performed by Pistorius *et al.* [10]. Here the absorption bands for lipid, protein and carbohydrate were baseline corrected and subsequently integrated (Figure 1a). A calibration of these integrals has been performed using single substance spectra of phosphatidyl choline for lipid, creatine phosphokinase for protein and malt extract from starch hydrolysate for carbohydrate.

Since Pistorius *et al.* [10] measured in transmission mode, similar procedures can also be applied to attenuated total reflectance (ATR) spectroscopy [11,17]. Mayers *et al.* [11] characterized 30 samples by biochemical methods and subsequently related the band integrals with the percentage of compound per dry weight. A least squares polynomial regression analysis has then be performed to calibrate the peak area against the macromolecular contents for a quantitative determination of cellular macromolecules. Laurens and Wolfrum [17] also used a Partial Least Square (PLS) regression analysis but for the determination of different lipids. These experiments were based on exogenously added lipid and showed an accurate prediction of added lipid levels for triglycerides and phospholipid. This work shows the application of mathematical methods to separate between different lipids, but the quantification of this lipid biomass will still need a true calibration.

However, both these methods do not take into account the overlap of vibrational bands from the different macromolecular pools. Both preferred different spectral regions for the calibration of the lipid content (2984–2780 cm^−1^ methyl and methylene groups or stretching of ester bonds at 1740 cm^−1^), which can be explained only by different cellular biochemical ratios. However, the overlap of other constituents is species-specific e.g., reference spectra of carbohydrate show a distinct peak at 2927 cm^−1^ which is in the range of the methyl bands used for lipid quantification. Absolute amounts of macromolecules will then be overestimated to some extent. This overlap of absorption bands holds true for at least all of the analyzed macromolecules (Figure 1b). Alternatively, a multiple linear regression protocol has been performed for fitting single reference spectra into the original cell spectra to calculate the amount of each macromolecule [13] minimizing this overestimation (Figure 1b). This spectra reconstruction also needs the calibration by reference substances. Wagner *et al.* [13] used triglycerol for lipid, bovine serum albumin for protein and glucose for carbohydrate as reference. Each of the single reference substance has been calibrated by correlating the absorption intensity *versus* the substance concentration. By a multiple linear regression algorithm, all reference spectra are then fitted into the cell spectra at once. The remaining error should then be as small as possible and is normally expressed as RMSE (root mean square error). The sum of the fitted reference spectra lead to a spectrum similar to the cell one. Furthermore, the residuals (*i.e.*, the differences between the original cell spectrum and the fitted one) can be used to identify absorption bands that are due to other IR active substances present in high amounts or characterized by high extinction coefficient. Since the reference spectra have been calibrated, the absorption of the fitted reference spectra can be used to quantify the contributing amount of the respective molecule into the cells.

However, both methods have shown the applicability of IR spectroscopy with some restrictions. The validity of Lambert Beers law has been evaluated by Wagner *et al.* [13] showing that sample thickness is a crucial parameter. Also attenuation of the infrared radiation across the sample pellet can influence an absolute IR spectroscopic quantification [16], so that the amount of probe material used has to be in a narrow range and handled with care [13]. Furthermore, the method is currently restricted to cells that do not have any silica shells, since silica absorption bands overlap with the absorption bands used for carbohydrate determination [13]. Nevertheless, if the C:N ratio of the biomass is known, also FTIR spectra of Si-containing diatoms can be analyzed [18,19]. Since FTIR-spectroscopy is still a newly developed method, several authors attempted to validate the measurements by independent techniques. Liu *et al.* [20] extracted the cellular lipids by methanol chloroform to confirm the band assignment of the lipids. Furthermore, they could show from the isolated lipid the amount of carbohydrate that is bound to the lipid acids. Since the absorption of the lipid band at 2926 cm^−1^ is much higher than the carbonyl absorption at 1740 cm^−1^ the authors suggest to use the band around 2926 cm^−1^ for quantifying the lipid content. They further used a normalization procedure by scaling the spectra to the amid I as well as amid II bands and could document that the normalization to the amid II band is more suitable than that performed on amid I. Meng *et al.* [21] validated their FTIR analysis by traditional biochemical methods, but explained only approx. 50%–70% of the dry weight. With the assumption that more than 90% of the biomass is composed of lipid, protein and carbohydrate, FTIR methods are still questionable with respect to absolute quantifications. However, the macromolecular contents measured via IR spectroscopy expressed as band area ratios correlated to the biochemically quantified macromolecular ratios.

In some cases it could be interesting to analyze the biochemical content and distribution at the single cell level. Powerful IR radiation gained from a synchrotron light source can open this possibility, thus one can obtain FTIR spectra with a spatial resolution of 5 µm to 10 µm [22,23]. However, new IR imaging techniques reach almost 0.54 µm of spatial resolution [24] but absolute quantitative measurements have not been published so far.

## 3. Application in Biotechnology

FTIR analysis can be a helpful tool in biotechnology especially for development of lipid producing phototrophic organisms by changing culture conditions or genetically engineering. Already existing bioreactors need a dense monitoring program to identify (i) contaminations and (ii) to assess the yield of the compound of interest.

The optimization of lipid production for biofuels for example needs fast and high throughput methods to monitor cellular macromolecular concentrations and lipid yields. Lipid quantification by means of FTIR spectroscopy has been shown to be a reliable method compared to the gravimetric determination [25,26], which needs milligrams of biomass that are hard to obtain in high throughput screening. For this reason several authors used FTIR spectroscopy to evaluate the lipid productivity and cell growth with respect to heterotrophic growth, different nutrient limitations and abiotic factors like light and temperature [16,20,26,27,28,29]. Under nitrogen limitation green algae cells change their cellular lipid and carbohydrate contents [20,30]. Using FTIR spectroscopy Liu *et al.* [20] could discriminate cells with higher lipid contents by a principle component analysis (PCA) analysis. This method can further be used to describe the trade-off between lipid content, growth rate and cell density as a result of the nutrient starvation. In *Chlamydomonas vulgaris* cultures it was shown that FTIR spectroscopy can be used to follow the overall carbon allocation pattern during different growth phases and under different nitrogen and CO_2_ availability [27]. The authors could demonstrate that high growth rate and high lipid accumulation can be obtained under different growth conditions. From these data they could calculate the lipid productivity per dry weight, from which the best time of harvesting can be obtained [27]. In *Nannochloropsis sp.* the response to organic solvents for lipid “milking” has been investigated [31]. Some solvents increased the total lipid content but FTIR spectroscopy has shown that the composition itself does not change. In conclusion, cell homeostasis can be monitored during an experiment by FTIR spectroscopy, even if the production rate of a specific compound is increased and excreted by the cell into the medium [31]. These examples showed the variety of applications of FTIR spectroscopy to monitor the effects of the culture conditions on the macromolecular response of the cells. Hence, FTIR it is now established to compare several species to optimize the lipid yield Jiang *et al.* [32], or to identify carbohydrate accumulating strains that are also potential bio-ethanol producers [33]. In conclusion FTIR spectroscopy is a fast and simple method for evaluating and monitoring culture conditions not only in bioreactors by providing information on the macromolecular content of cells [28,34].

Besides these monitoring of culture condition dependent accumulation of lipid and carbohydrate, FTIR spectroscopy can be applied for screening of new developed mutants with altered biochemistry. An example is the analysis of the biochemical pathway of carbon assimilation via C4 metabolism in diatoms. It has been hypothesized that mutants of a silenced PPDK would have a higher lipid accumulation. This hypothesis could be confirmed by chemometric analysis of IR spectra [35]. Finally, not only lipid accumulation, but the whole cellular macromolecule ratios can be useful for biotechnology. Mutants of cyanobacteria are potentially useful for hydrogen production. Therefore, different Synechocystis strains have been modified in antenna size, leading to altered phycobillisomes and increased electron transport rates. These patterns can then be monitored by FTIR to document the metabolic changes in mutants compared to wildtype cells [36].

## 4. Physiology and C-Dynamics

FTIR spectroscopy can be used for the optimization of phototrophic organism performance in biotechnology. Thereby it shows the steady state measurements of the macromolecular pools. However, this method can also be used to evaluate physiological responses within a variety of applications, ranging from whole cell physiology [37,38] to responses on a very small molecular scale, e.g., the response to down regulation of single genes [35]. In addition measurements can be performed on different time scales ranging from days down to several minutes.

Since the FTIR spectroscopy reflects all cellular macromolecules, a measure of stored energy or an energy balance of the biomass can be determined [37]. In more detail, measurements of energy balance are generally performed via gas exchange or electron transport rates based on fluorescence measurements. However, for detailed information of the cellular energy distribution, the electron allocation into different carbon pools has to be considered. There are two main physiological strategies concerning carbon allocation, especially under changing growth conditions. Either cells stay in a very stable homeostatic macromolecular composition [39] or a significant change of macromolecule ratios can be measured [19]. Nevertheless, for almost all physiological experiments FTIR spectroscopy can deliver reliable information about the cellular carbon distribution and energy content. This has been shown by a number of publications ranging from acclimation to N, Si and Fe deficiencies [19,22,40] or cold temperature acclimation [41] up to different CO_2_ levels [27]. As pointed out before, monitoring of such physiological responses can be done with a timescale of several minutes. In *Chlamydomonas reinhardtii* grown in a 13 h/11 h light/dark cycle the carbohydrate to protein ratio rises up to 2 fold in the first four hours of the light phase showing that the newly formed photosynthates are directed to the carbohydrate pool, whereas later on this ratio remains constant indicating that carbon is funneled equally to carbohydrate and protein. During the dark period the carbon stored in the carbohydrate pool is reallocated to protein synthesis [13]. This shows that carbon allocation is highly regulated and a time series of FTIR measurements as biochemical snap shots can resolve this dynamics to define the best time point of harvest for special application. Based on the knowledge of these dynamics the more time consuming and costly methods with higher resolution for the detection of molecular compounds (such as mass spectroscopy based metabolomics) can be applied more efficiently.

**Figure 2:**
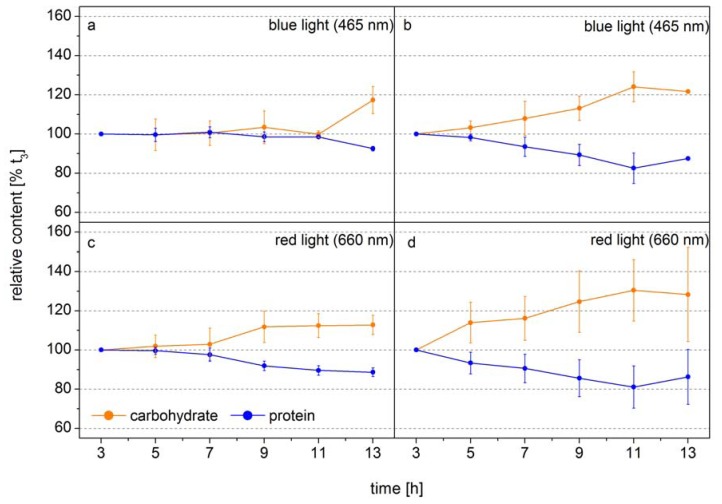
Carbohydrate (orange) and protein (blue) levels of *Phaeodactylum tricornutum* grown at blue light (465 nm) of (**a**) 10 µmol absorbed photons m^−2^ s^−1^ (Q_Phar_) and (**b**) 30 µmol absorbed photons m^−2^ s^−1^ or at red light (660 nm) of (**c**) 10 µmol absorbed photons m^−2^ s^−1^ and (**d**) 30 µmol absorbed photons m^−2^ s^−1^ in a 14 h light/10 h dark cycle. The time axes correspond to the time in the light phase. FTIR spectra were detected every 2 h and used for the calculation of the macromolecular composition (according to Wagner *et al.* 2010). The carbohydrate and protein levels were normalized to the values at 3 h into the light phase (t_3_) (modified according to [42].

The following example shows how FTIR spectroscopy can be used to identify the time point of metabolic switches in cells. In this experiment the diatom *Phaeodactylum tricornutum* was grown at two different light intensities in monochromatic blue light (BL, 470 nm) and red light (RL, 650 nm), to analyze the function of the blue-light receptors in diatoms [42]. The design of this experiment allowed the authors to obtain equal photosynthetic rates per cell by adjusting the incident RL and BL. Thus, changes in the macromolecular composition only related to light color modulated changes in the carbon allocation pattern. Figure 2 shows the relative cellular protein and carbohydrate contents. Under low BL light carbon is distributed equally into protein and carbohydrate during the whole light period and only at the end of the light phase the carbon flows to a higher proportion to the carbohydrate (Figure 2a). In low RL this carbon distribution pattern is observed only for seven hours, after that the carbon is preferentially diverted to carbohydrate (Figure 2c). Under high light, when the carbon flux produced from photosynthesis is higher, the new carbon is preferentially directed to carbohydrate after 3–5 h (Figure 2b,d). This shows that the carbon allocation pattern depends on both the flux and the photoreceptor controlled activities of enzymes directing the carbon to different pathways. The dynamics show that well-defined sampling for more detailed analysis by mass spectroscopy based metabolomics can be efficiently assisted by FTIR-spectroscopy by identifying the transition phase when the carbon flux direction is changing. If the overall carbon flux can be measured, as it is the case of photosynthetic active cells either by means of Chla *in vivo* fluorescence or CO_2_ uptake, the absolute flux into the different carbon pools can be quantified. Together with the semi-quantitative information of key metabolites obtained from mass spectroscopy analysis the dynamics in flux control in the metabolic network can be displayed in quantitative terms. This shows that traditional gas flux measurements, FTIR spectroscopy and mass spectroscopy based metabolomics can synergistically act to display a metabolic network picture of life.

## 5. Conclusions and Perspectives

FTIR spectroscopy delivers a fingerprint of the macromolecular composition of cells. Recent advances in chemometrics allow the quantification not only of major cellular pools like lipid, carbohydrate and protein but also minor components of interest. However, the resolution of complete metabolite pattern with quantitative or semi-quantitative estimates is not possible and is not in perspective. Compared to the high resolution of metabolome analysis, FTIR spectroscopy allows fast analysis with minimal amounts of material with the additional advantage of high throughput. This opens the perspective of time-resolved analysis which allows us not only to measure pattern but also fluxes. This potential is useful for biotechnological process control and to assist a well-defined sampling for mass spectroscopy based metabolomics.
